# Effect of Size, Shape and Surface Functionalization on the Antibacterial Activity of Silver Nanoparticles

**DOI:** 10.3390/jfb14050244

**Published:** 2023-04-26

**Authors:** Arianna Menichetti, Alexandra Mavridi-Printezi, Dario Mordini, Marco Montalti

**Affiliations:** Department of Chemistry “Giacomo Ciamician”, University of Bologna, Via Selmi 2, 40126 Bologna, Italy

**Keywords:** multi drug resistance (MDR), antibacterial, nanoparticles, antimicrobial, wound healing

## Abstract

Silver nanoparticles (AgNPs) are the most investigated antibacterial agents against multidrug resistant (MDR) pathogens. They can lead to cellular death by means of different mechanisms, damaging several cell compartments, from the external membrane, to enzymes, DNA and proteins; this simultaneous attack amplifies the toxic effect on bacteria with respect to traditional antibiotics. The effectiveness of AgNPs against MDR bacteria is strongly correlated with their chemical and morphological properties, which influence the pathways involved in cellular damage. In this review, AgNPs’ size, shape and modification by functional groups or other materials are reported, both to investigate the different synthetic pathways correlated with nanoparticles’ modifications and to evaluate the related effect on their antibacterial activity. Indeed, understanding the synthetic conditions for obtaining performing antibacterial AgNPs could help to tailor new and improved silver-based agents to combat multidrug resistance.

## 1. Introduction

Bacterial diseases are one of the main issues for human health; already in 2019, they represented the second top cause of death [1]. In recent years, the major concern linked to microbial infections is antibiotic resistance. The World Health Organization claimed that, for this reason, infections such as pneumonia, blood poisoning and foodborne illnesses are more and more difficult to treat [2]. Indeed, since the introduction of antibiotics in the 20th century [3], bacteria have evolved by developing defence mechanisms that reduce or deactivate antibiotic effectiveness. Drug resistance derives from many complex mechanisms of action [4], such as an increase in efflux pump expression, which removes drugs from the bacterial cell; genes that alter the binding substrate, modifying the drug targets; and proteins and enzymes that lead to drug alteration [5,6]. Typical multidrug-resistant pathogens include *Escherichia coli*, *Staphylococcus aureus* and *Pseudomonas aeruginosa* [7]. Another strategy adopted by bacteria in defence against antibiotic activity is the formation of biofilms [8]. A bacteria biofilm consists of a “structured community of bacterial cells enclosed in a self-produced polymeric matrix and adherent to an inert or living surface”, which allows bacteria to survive in hostile conditions [9]. They can exchange nutrients by channels and express different genes in different biofilm areas [10,11]. Biofilm spatial heterogeneity is one of the causes of antibacterial resistance, as it allows part of the biofilm to survive an antibiotic attack [9,12,13]. Another strategical property of biofilms is the difficulty antibacterial agents have penetrating through such an ordered structure; antimicrobials diffuse slower in biofilms than in water [9,14], making bacteria biofilms able to tolerate antibiotic concentrations up to 1000 times higher than planktonic bacteria can [5]. Nowadays, MDR represents the main challenge faced in antibacterial agents development, not just as a risk for human health, but also as a huge concern for healthcare system costs [15,16]. Consequently, there is an urgent need to find alternative methods that can efficiently treat bacterial infections, and nanomaterials are possible candidates for antibacterial activity.

### 1.1. Silver Nanoparticles as Antibacterial Agents

**Figure 1:**
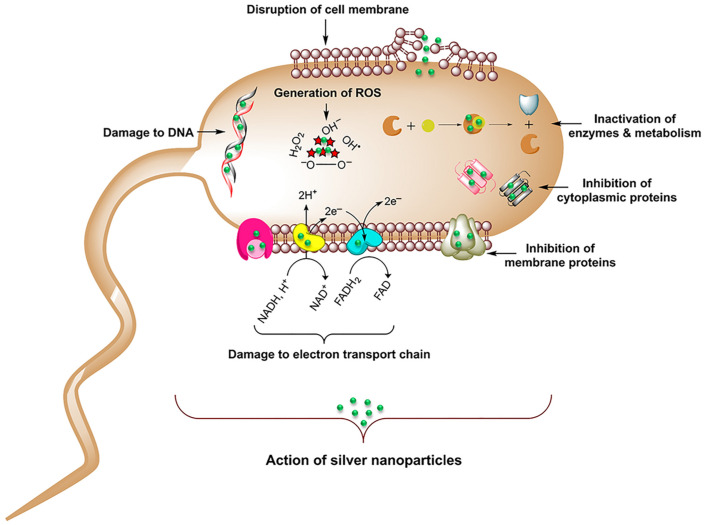
Representation of the possible pathways of cellular damaging by AgNPs. Adapted with permission from ref. [45].

In the last 15 years, researchers have deeply investigated the properties of nanoparticles for biomedical applications [17]; these can be metallic nanoparticles [18], such as silver and zinc [19], as well as metalloids [20], and even organic/inorganic compounds [21,22]. Indeed, due to their size, nanoparticles can be internalized in cells via mechanisms that are different with respect to small molecules [23], and that can change based on the physiochemical parameters of the nanoparticles [24,25,26,27]. Additional beneficial characteristics of nanoparticles’ application in the biomedical field include biocompatibility [28,29], specific target recognition [24] and the ability to functionalize the surface and use them as carriers for drug delivery [30,31]. Most of the research attention in antibacterial activity has been focused on the development of silver nanomaterials. In fact, silver has been used as an antibacterial agent for thousands of years, ranging from food preservation and water sanitization in early civilization [32,33] to healing burn wounds [34]. Silver nanoparticles (AgNPs) have been extensively studied, and they have been reported to have an effect against both Gram-negative and Gram-positive bacteria, although Gram-positive bacteria are less sensitive to the action of AgNPs than Gram-negative ones [35,36,37]. This is attributed to the difference in the Gram-negative and Gram-positive bacteria surfaces: Gram-negative bacteria have a thin cell membrane (8–12 nm) with negatively charged lipopolysaccharides, promoting nanoparticles adhesion [38], while Gram-positive bacteria, on the other hand, have a thicker membrane (20–80 nm) and negatively charged peptidoglycans that can be an obstacle for AgNPs’ penetration [35,39,40]. Indeed, the main action of AgNPs in killing bacteria is interacting with and modifying the external bacterial membrane. Once accumulated on the surface, they manage to cause damage to the membrane by penetrating and perturbing the membrane’s permeability [15,41]. Following the penetration in the cell membrane, AgNPs can also interact with and damage the internal cellular compartments, affecting vital functions [42,43,44,45] (Figure 1). However, the clear mechanism of interaction that makes AgNPs so efficient is still not clear due to the different processes competing in AgNPs activity.

### 1.2. Antibacterial Mechanisms of Silver Nanoparticles

A complete overview of the mechanisms involved in AgNPs’ interaction with bacteria is given by Le Ouay and Stellacci [46]. Their work elucidates the role of the Ag^+^ species, which is the main one responsible for the antibacterial activity. Indeed, Ag^+^ has the ability to easily bind to amines, phosphates and thiols [47], common biological functionalities, and also to interact with DNA and peptides. Considering its low selectivity, it is hypothesized that Ag^+^ interacts with several targets simultaneously, favouring bacteria death. Thus, the action of perturbing the bacterial membrane derives from the generation of Ag^+^ from the oxidative dissolution of AgNPs. This is the reason why the size of the nanoparticles has an impact on the antibacterial activity: a smaller nanoparticle dissolves faster, releasing more Ag^+^. Another relevant toxicity factor responsible for AgNPs’ antibacterial activity is the generation of reactive oxygen species (ROS), which leads to cell oxidative stress and apoptosis [44,48]. The increase in ROS triggered by AgNPs is governed by two mechanisms. The first one relies on the presence of reactive groups, such as radicals or oxidants, that are often present on the surface of the nanoparticles and directly act in the ROS formation [49,50,51]. The second one involves the deactivation of the ROS protection pathways, such as glutathione (GSH-typical cell antioxidant) damage, eliminating the scavenging activity against ROS formation [52]. It is evident that, as in the case of the Ag^+^-related mechanism, ROS generation is also driven by nanoparticles’ properties, such as the size and shape. Indeed, even though the precise toxicity pathways are still unclear, it can be said that they seem to be correlated with surface properties.

Therefore, control of the size, shape and surface functionalities of nanoparticles is pivotal for the design of a highly efficient Ag-based antimicrobial agent. In this review, we will analyse how the size, shape and combination of AgNPs with other materials can impact their antibacterial performance against the most common MDR pathogens. At the moment, there are multiple synthetic methods to obtain antibacterial AgNPs, and each of them can lead to different chemical and morphological parameters that affect nanoparticles’ behaviour. Moreover, recent interesting examples of AgNPs-based materials already designed for biomedical applications will be reported. In this regard, the aim of this review is to provide guidelines for the improvement of new AgNPs-based systems, towards a more effective antibacterial performance against drug-resistant microbes, which is a matter of increasing urgency for the scientific community.

## 2. Size-Dependent Effects

**Figure 2:**
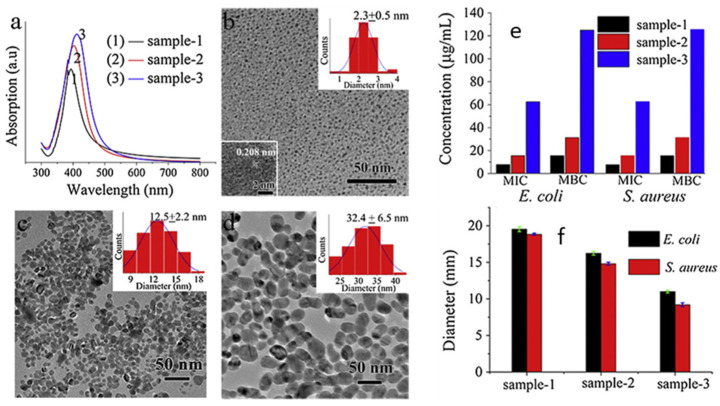
(**a**) UV-Vis and TEM characterization of AgNPs: (**b**) at pH 11 (sample 1), with HRTEM image inset; (**c**) at pH 9 (sample 2); (**d**) at pH 7 (sample 3). Particle size distribution insets are reported in (**b**–**d**). (**e**) MIC and MBC values and (**f**) diameter of inhibition zone of samples against *E. coli* and *S. aureus*. Adapted with permission from ref. [53].

Size is the main parameter that determines the efficacy of AgNPs’ antibacterial activity, affecting both the intrinsic AgNPs activity and the cellular uptake. Indeed, many synthetic methods are performed in order to obtain size control. In this section, we will provide an overview of the most common methods to obtain AgNPs of different size and their effect on antibacterial activity and the mechanism. A first example of size control is given by Wu et al., who reported the synthesis of AgNPs using sodium borohydride (NaBH_4_) as the reducing agent and sodium citrate as a stabilizing agent [53]. They modulated nanoparticles’ size by modifying the pH of the reactant solution, obtaining sizes of 2 nm, 12 nm and 32 nm at pH 11, 9 and 7, respectively, characterised by UV-Vis, TEM (Figure 2) and XRD. The NPs, tested with Gram-negative *Escherichia coli* (*E. coli*) and Gram-positive *Staphylococcus aureus* (*S. aureus*) bacteria by means of the minimum inhibition concentration (MIC), minimum bactericidal concentration (MBC) and inhibition zone diameter, displayed good antibacterial properties, which increased with the particle size decrease. Indeed, 2 nm nanoparticles showed the best performances in all the tests, as observed in Figure 2. Chen et al. [54] proposed antibacterial AgNPs for wound healing, synthesizing them with a physical method. They used an evaporation–condensation system, exploiting evaporation at high pressure and condensation under low temperature [55]. This method allowed obtaining silver particle populations with very small size, ranging from the nanometre to the Ångstrom scale. They embedded particles of about 6.6 nm (65.9 ± 31.6 Å), named “L-AgÅPs”, in a carbomer geland and verified the distribution using SEM-EDX, TEM, XRD and FTIR. The antibacterial activity was evaluated against Gram-positive *S. aureus* bacteria and Gram-negative *Pseudomonas aeruginosa* (*P. arginosa*) in vitro, as well as against biofilm formation. The L-AgÅPs were compared with larger commercial AgNPs embedded in the same carbomer gel. In all the tests performed—from MIC and MBC to optical density (OD) for the biofilm—the L-AgÅPs gel demonstrated far better results than the commercial AgNPs counterpart gel. The same result was also achieved when comparing the behaviour in the topical application of the gel on mouse skin, which revealed a relevant effect of the nanoparticles’ sizes on the antibacterial performances. Although chemical and physical approaches for AgNPs size control are successful most of the time, these techniques are often performed in extreme conditions, involving toxic agents and using very expensive equipment. For these reasons, researchers have recently focused on biological pathways, obtaining cheaper and more environmentally friendly methods that manage to exert size control.

Skandalis and co-workers [56] used the leaf extract of the plant *Arbutus unedo* as a reducing and stabilizing agent to synthesize AgNPs with two different sizes, 58 nm (standard deviation 18.4%) and 40 nm (standard deviation 33.6%), by tuning the amount of leaf extract during the process. The antibacterial activity was evaluated by MIC and MBC on *B. subtilis*, *S. epidermidis*, *E. coli* and *P. aeruginosa*, showing the good antibacterial performance of the AgNPs, independently of the size. However, a difference between the 58 nm- and 40 nm-sized nanoparticles was highlighted when comparing the effects of the two systems in the interaction with *E. coli*, studied by SEM, after 5, 10 and 24 h. While in the case of 58 nm nanoparticles, the bacteria membrane showed disruption after 24 h of interaction, 40 nm nanoparticles managed to break the membrane already after 10 h, displaying a stronger effect on bacteria inhibition.

Another example of plant derivatives as reducing and stabilizing agents was presented by Balu et al. using *Rosa Indica* petal extracts [57]. In this case, AgNPs with different sizes were tested by changing the solvent of the extraction (12 or 18 nm using acetone or ethanol respectively, and 770 nm using water), and as expected, the smallest nanoparticles showed the highest antibacterial properties against both *E. coli* and *S. aureus*.

Green-synthesized AgNPs were also described by Hileuskaya and co-workers, who used pectin as a stabilizing agent and obtained different sizes based on the pectin esterification degree and functional groups [58]. TEM characterization revealed nanoparticles ranging from 8 to 13 nm in all the prepared samples, apart from the ones capped by amidated low methoxy pectin (PectA_Ag 25:1), which had a size of 28 ± 7 nm. The antibacterial activity, evaluated by the dilution assay and well diffusion method, against Gram-positive *Bacillus* (*subtilis* and *pumilus*) and Gram-negative *E. coli* was enhanced for smaller, rather than bigger, nanoparticles.

Polymers are also common capping agents and can be used to tune nanoparticle sizes, as shown in the research of Ji et al., who obtained ultrasmall AgNPs with a thermosensitive copolymer [59]. They obtained three different AgNPs of 3.91 nm, 2.29 nm and 1.59 nm (characterized by TEM), deriving smaller nanoparticles by increasing the polymers’ quantity with respect to AgNO_3_. The antibacterial activity against *E. coli* and *S. aureus* was studied by means of MIC, MBC and optical density analysis to monitor the copolymer behaviour at different temperatures. The results showed that while the smallest nanoparticles had the best antibacterial performance at 28 °C, an inverted trend was observed at 37 °C. The higher temperature caused the copolymer to collapse, preventing the interaction with bacteria. This effect was stronger in the smallest AgNPs that were synthesized with the highest polymer content, and this example highlights the influence of the AgNPs’ surface properties. Another example of the combination of size and surface properties in the antibacterial activity of AgNPs is reported by Haidari et al. [60], who synthesized ultrasmall (<3 nm) AgNPs functionalized by chitosan to obtain a polycationic surface. These nanoparticles presented very good antibacterial and antibiofilm properties, in comparison with AgNPs of the same size with a negatively charged surface.

As size-dependent cellular transport and accumulation are still controversial, research has recently focused on studying the cellular uptake mechanisms in correlation with nanoparticles’ size. For example, Fernández et al. evaluated the size effect of AgNPs’ cytotoxicity by means of the behaviour of a set of proteins [61]. AgNPs of 10 nm and 60 nm were compared in the internalization mechanism of hepatic (HepG2) cells, chosen because of the frequent AgNPs accumulation in the liver. First, the authors noted the different nanoparticle distribution, in which the 10 nm NPs managed to penetrate in the nucleus, while the 60 nm ones aggregated in the cytoplasm. Then, a quantitative proteomic analysis was performed, revealing almost 50 altered proteins for both the 10 nm and 60 nm AgNPs. However, among these proteins, only four were deregulated in the same way by the two nanoparticles, meaning that the mechanisms that involved all the other proteins, even if they often led to the same result, followed completely different pathways, depending on the nanoparticles’ size only. Wu et al. focused instead on the mechanism of cellular uptake based on AgNPs of 3.2 ± 0.5 nm, 20.7 ± 2.5 nm, 54.7 ± 7.7 nm and 93.6 ± 9.6 nm [62]. They investigated the size dependence on the internalization pathways in a mouse melanoma cell line (B16), observing that AgNPs, based on the size, not only influenced the uptake efficiency, but also followed different types of endocytosis.

The control over silver nanoparticles’ size is of wide interest because it is one of the parameters that mostly influences their antibacterial properties. Indeed, among the several methods that could be performed to obtain size control, the same trend in the size-dependent efficiency is usually reported: the more the size decreases, the more the bacteria inhibition activity increases. In fact, any variation of this trend could just be due to different surface properties of the AgNPs, which can influence their interaction with the bacterial membrane, as will be discussed later.

Size control is not only relevant to investigating the AgNPs’ antibacterial activity, but also to better understand their toxicity [63]. As a matter of fact, cytotoxicity is another relevant issue that has to be considered when evaluating the antibacterial activity related to biomedical applications, and that will be discussed in the sixth section of this review.

## 3. Shape-Dependent Effects

**Figure 3:**
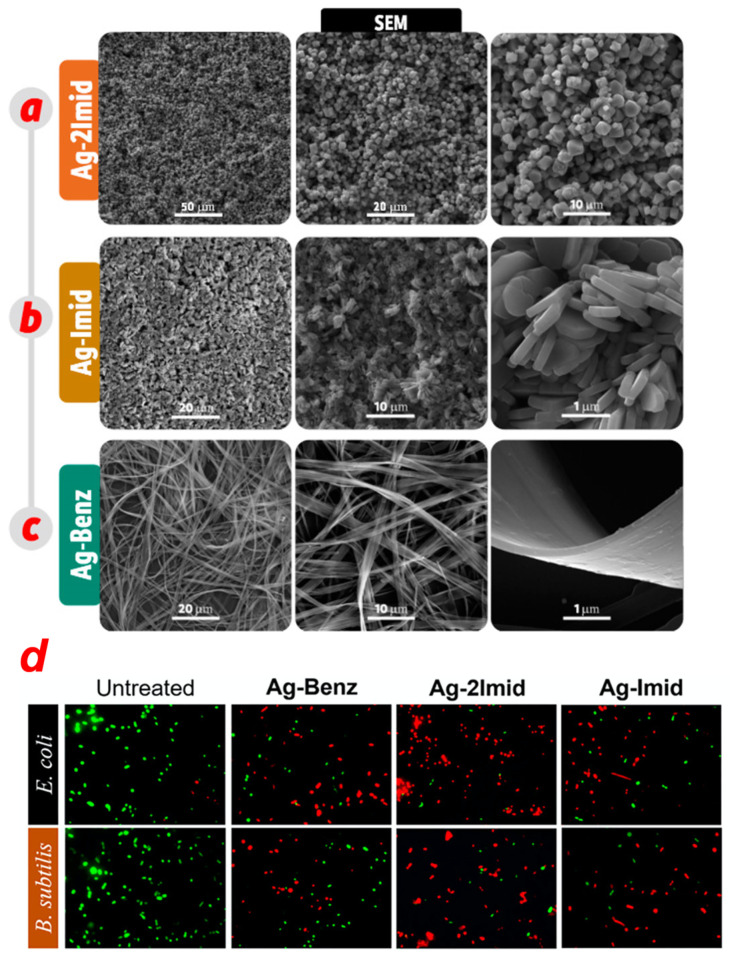
FE-SEM images of (**a**) Ag-2Imid, (**b**) Ag-Imid and (**c**) Ag-Benz at different magnifications. (**d**) Antibacterial activity of Ag-MAFs against *E. coli* and *B. subtilis* observed with fluorescence images of the samples stained with propidium iodide/SYTO9. Adapted with permission from ref. [71].

Beyond size, shape also plays an important role in the antibacterial performance of AgNPs. Non-spherical AgNPs can be obtained with several methods, which range from chemical methods to bio-based synthesis. The antibacterial effects of differently shaped AgNPs have also been investigated, and the antibacterial activity based on shape depends on how good the contact is between the nanomaterial and the bacteria cell membrane. A very clear example of this is given by Hong and co-workers [64], who compared the antibacterial properties of Ag nanospheres, nanocubes and nanowires. These were obtained by AgNO_3_ reduction in the presence of ethylene glycol, polyvinylpyrrolidone (PVP) and NaCl, using microwave-assisted synthesis. The obtained nanomaterials were then characterized by UV-Vis spectroscopy, TEM and XRD, showing cubic, spherical and wire morphologies with different crystal facets. The antibacterial activity was investigated on *E. coli* by the optical density method (OD) growth curve test and the minimum inhibitory concentration (MIC) test. All the AgNPs’ shapes presented antibacterial properties. The cubic-shaped nanomaterials demonstrated the best antibacterial performances, followed by the nanospheres and the nanowires. The weak activity of the nanowires can be explained by observing the nanomaterial interaction with bacteria by TEM, which showed that nanospheres and nanocubes had closer contact with the bacteria surface, as opposed to nanowires. However, different activity was also reported when comparing nanocubes and nanospheres and the crystal facets of the two morphologies. The nanocubes’ facet (100) had a higher surface energy than the (111) facet of the nanospheres, leading to a higher reactivity [65,66,67]. An interesting study on the shape-effect activity of AgNPs was proposed by Goyal et al. [68]. They synthesized silver nanomaterials, starting from AgNO_3_ and PVP, by means of a solvothermal method. The nanoparticles were characterized by absorption spectroscopy, XRD and TEM, and the antibacterial activity was evaluated for *E. coli*, *V. cholerae*, *bacillus*, *S. aureus* and *S. pyogenes*, using the standard well diffusion method in Mueller–Hinton agar (MHA) plates [69]. All the samples tested were plate-like nanoparticles with different behaviours based on their edges. The authors compared triangular (prismatic)/hexagonal nanoparticles of the same size, but with different edges (sharp or round), and observed higher antibacterial activity for the sharpest ones. The reason was that the higher charge density in sharp edges, when compared with the round ones [70], can lead to a higher disturbance of the permeability of the bacteria membrane, causing an easier rupture. Another morphologic comparison of the antibacterial activity is given by Seyedpour and co-workers [71]. They investigated the antimicrobial performance of silver-based metal–azolate frameworks (Ag-MAF). In this case, the authors synthesized supramolecular structures, using imidazole, 2-methylimidazole, benzimidazole and Ag^+^ as coordination polymers, obtaining Ag-MAF, Ag-Imid, Ag-2Imid and Ag-Benz, respectively. The structures were then characterized by UV-Vis, TEM, SEM and XRD, and three different morphologies were observed: octahedral for Ag-2Imid, hexagonal for Ag-Imid and nanoribbons for Ag-benz (Figure 3a–c). Next, XPS was performed, showing the following order for the silver atoms content: Ag-2Imid > Ag-Imid > Ag-Benz. The antibacterial activity was investigated using propidium iodide (PI), whose fluorescence is enhanced when intercalated into double-stranded DNA [72] and can be switched on in a damaged cytoplasmatic membrane and using a SYTO9 fluorescence probe, which can be internalized, even in intact bacteria membranes. In this way, the authors were able to evaluate the ratio of dead and live cells (Figure 3d). Tests were performed on *E. coli* and *B. subtilis* and resulted in antibacterial activity of the order Ag-2Imid > Ag-Imid > Ag-Benz.

In these systems, many parameters could have influenced the antimicrobial performance, such as ligands antibacterial properties and the Ag^+^ content and morphology. As far as concerns the last, the higher activity of Ag-2Imid in this case was correlated with the sharper edges of the octahedral structures, which allowed closer interaction and better permeation in the bacteria membrane.

Cheon et al. [73] synthesized spherical, disk-like and triangular plate AgNPs and characterized the three different morphologies by UV-Vis and TEM. The antibacterial activity was evaluated using the disk diffusion and OD methods against *E. coli*, *S. aureus* and *P. aeruginosa*. In contrast with the examples cited above, spherical nanoparticles in this case exhibited the highest antibacterial activity with respect to the other shapes. The authors calculated the surface areas of the three nanoparticles and found that the spherical shape had the biggest surface area, leading to higher reactivity and a higher Ag^+^ release rate, which was evaluated by ICP. Moreover, it must be taken into account that the nanospheres were smaller (ca. 38 nm) than the nano-disks/nano-triangles (ca. 50 nm), and thus, the size effect could have also had a role in the antibacterial activity.

As for the size control, plant extracts have also been used to control nanoparticles’ shape. Salayová and co-workers [74], for instance, conducted AgNPs synthesis from five aqueous plant extracts, influencing both the size and morphology. The nanoparticles were characterized by UV-Vis, TEM, XRD and FTIR. Based on the plant extract, nanoparticles ranging from 15 nm to 75 nm in size were obtained with spherical (for the smaller nanoparticles) and truncated octahedron morphologies (for the larger ones). Another example of plant-based shape control of AgNPs synthesis is given by Parit et al. [75], who used the extract of the *G. rensifera* medicinal plant. Nanoparticles were obtained by mixing AgNO_3_ and *G. rensifera* extract, and the size and shape were modified based on the AgNO_3_ concentration. At a low AgNO_3_ concentration, smaller (13 nm ± 5 nm) and prevalently spherical nanoparticles were obtained. The increase of AgNO_3_ concentrations led to the formation of larger-sized hexagonal (40 nm ± 22 nm) and triangular (prismatic) (44 nm ± 26 nm) nanoparticles. In all of the above cited works that propose the plant-based synthesis of AgNPs, no specific comparison of the activity of the different nanoparticle shapes was reported. Indeed, in both cases, it was not possible to control the nanoparticles’ size, making it more difficult to perform any shape-based comparison of their antibacterial properties.

When comparing different shapes of AgNPs, it is possible to individuate the following four morphology parameters that influence their antibacterial properties: crystal facets, sharpness of the edges, geometry of interaction and surface area. The different shapes are characterized by different crystal facets and reactivities. Moreover, sharp edges morphology, as explained above, can facilitate the interaction with the bacteria membrane and contribute to its destabilization. The presence of a geometry that physically allows closeness between the nanomaterial surface and the bacterial surface is also important. Spherical, triangular and octahedral objects can be attached more closely to the bacteria membrane than wires. Finally, the geometries that lead to better antibacterial activity are the ones with the highest surface area.

## 4. Silver Nanoparticles Functionalized by Biomolecules

**Figure 4:**
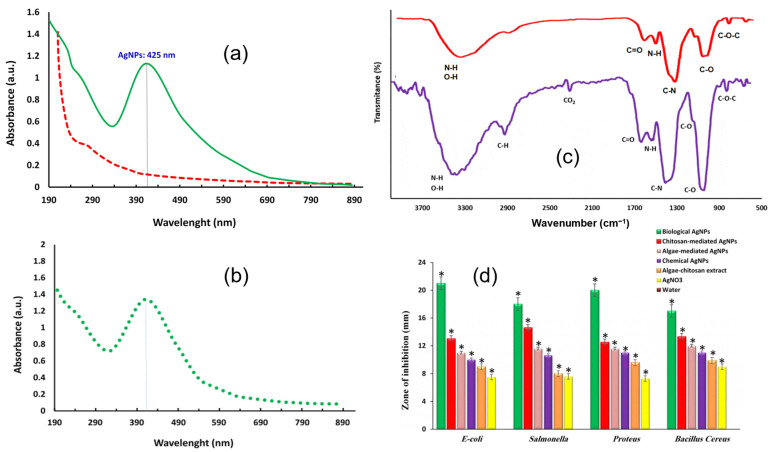
(**a**) UV-Vis spectra of chitosan-algae AgNPs (green) compared to chitosan-algae extract (dashed red). (**b**) Chitosan-algae AgNPs’ stability after six months. (**c**) FTIR spectra of chitosan-algae extract (purple) and chitosan-algae AgNPs (red). (**d**) Bacteria inhibition effect of chitosan-algae AgNPs (biological AgNPs) compared to chitosan-mediated AgNPs, algae-mediated AgNPs, chemical AgNPs, algae-chitosan extract and AgNO_3_. (*) Error bar. Adapted with permission from ref. [78].

In the optics of optimising the antibacterial performances of AgNPs, research focuses on achieving the best nanoparticles–bacteria interaction, enhancing biocompatibility and conducing the synthesis in mild conditions. For these reasons, among the possible synthetic pathways, greater attention is given to the biological synthesis of AgNPs, rather than physical or chemical methods. Indeed, the main investigated synthetic procedures concern the use of plant extracts as reducing and capping agents for silver nanomaterials; medicinal plants are often exploited, coupling the antibacterial effects of both the plant extract and silver. For example, Khan and collaborators [76] obtained gold and AgNPs using *Clerodendrum inerme* (medicinal plant) leaf extract. Morphological characterization was performed by TEM and XRD, reporting 5 nm-sized crystalline AgNPs. FTIR was used to verify the presence of *C. inerme* functional groups on the nanoparticle surface, confirming their presence. The antibacterial activity was studied against bacteria, *B. subtilis* and *S. aureus* (Gram-positive) and *Klebsiella* and *E. coli* (Gram-negative), and fungi, *A. flavus* and *A. niger*, by means of the zone of bacteria inhibition (ZOI) and MIC. *C. inerme* AgNPs displayed higher antimicrobial activity than commercial AgNPs (functionalized with PVP); the same results were obtained for biofilm inhibition. FTIR was also used to verify the interaction between nanoparticles and microbial cells, showing changes in fatty acids and protein structure related to cell membrane disruption. Another example of AgNPs green synthesis is given by Garibo et al. [77], who used a medicinal tree extract, *Lysiloma acapulcensis*, whose molecular components could act as reducing agents and stabilizers. Crystalline nanoparticles with a size ranging from 1.2 nm to 62 nm were characterized by TEM, XRD, SEM-EDS and SAED. The antibacterial activity was evaluated, with MIC and minimum biocidal concentration (MBC), against Gram-negative *E. coli* and *P. aeruginosa* and Gram-positive *S. aureus* and *C. albicans*, reporting significant activity, but at lower concentrations than AgNPs obtained by chemical methods. Rezazadeh et al. [78] instead decided to combine polyphenolic groups and chitosan, both obtained from marine organisms and both acting as reducing and capping agents in AgNPs biosynthesis. The obtained crystalline AgNPs with a size of almost 12 nm (verified by DLS, TEM and XRD) presented a high stability, even over months, monitored by absorption spectroscopy (Figure 4a,b). FTIR analysis verified the presence of both polyphenol compounds and chitosan, other than alkaloids, polysaccharides and proteins (Figure 4c); TGA further confirmed AgNPs’ functionalization by these biomolecules. The antibacterial activity was studied by the disk diffusion method against Gram-negative *E. coli*, *Proteus* and *Salmonella* and Gram-positive *Bacillus cereus*. As reported above, when comparing Gram-negative and Gram-positive bacteria, the former presents the highest inhibition of bacterial growth. However, these biological nanoparticles present a superior antibacterial performance with respect to the chemical-made counterparts, thanks to their higher bioavailability (Figure 4d).

**Figure 5:**
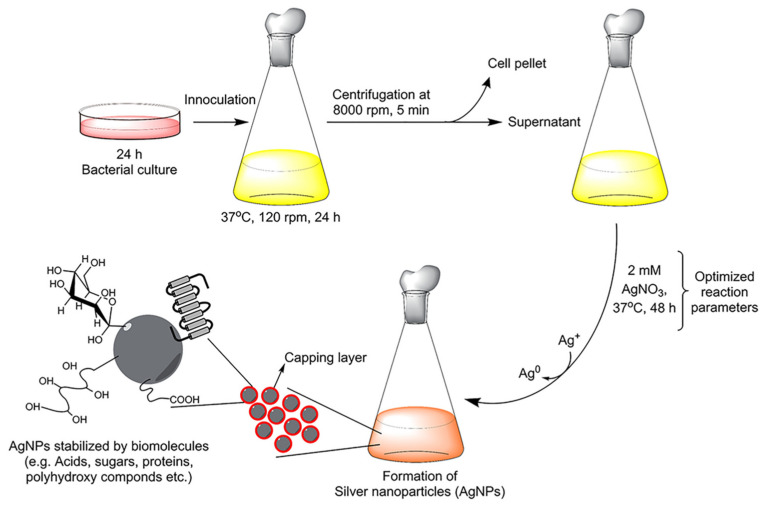
Procedure scheme of AgNPs synthesis in the presence of *Cedecea* sp. strain. Reprinted with permission from ref. [45].

In an alternative to plant extract, bacteria-derived biomolecules have also been explored for AgNPs synthesis. In particular, there are two methods for this kind of synthesis, namely, intracellular and extracellular. The mechanism of silver reduction by bacteria, even if still obscure, consists of metal ions trapping on the surface of or inside the bacteria and reduction by enzymes [79]. Extracellular synthesis is performed only in the presence of the supernatant of a bacteria culture and is usually more convenient because it requires fewer purification steps [80]. In the work of Singh et al. [45], AgNPs with the *Cedecea* sp. strain were obtained by means of a extracellular method: bacteria culture supernatant was mixed with AgNO_3_ and incubated for 1–2 days (Figure 5). TEM, SAED, DLS and AFM showed crystalline nanoparticles of 10–40 nm. FTIR confirmed the presence of amino acids, proteins and other biomolecules found already in the *Cedecea* supernatant. The antibacterial activity was investigated by MIC, MBC and the biofilm inhibition of *E. coli*, *P. aeruginosa*, *S. aureus* and *S. epidermidis.* The AgNPs exhibited effective antibacterial activity against the Gram-negative *E. coli* and *P. aeruginosa*, but they displayed weaker activity against Gram-positive *S. aureus* and *S. epidermidis*. An example of intracellular bacteria-mediated AgNPs synthesis was presented by Ahmed and co-workers [81], who used the *Bacillus safensis* (*B. safensis*) strain. Characterization with UV-Vis, TEM, SEM, XRD and FTIR confirmed the presence of AgNPs of 23–46 nm, which were functionalized by biomolecules present in the bacterial culture. The nanoparticles presented good antibacterial activity, evaluated by the disk diffusion method (MIC), against both *E. coli* and *S. aureus*. An interesting alterative to plant/bacteria-derived AgNPs, which is able to enhance their biocompatibility, is to synthesize and functionalize them with DNA.

The advantages of DNA are its biocompatibility and its molecular recognition ability [82]; moreover, DNA, thanks to its affinity for metal cations, is a suitable candidate for metal nanoparticles preparation [83,84]. For example, Liu et al. synthesized and investigated the antibacterial activity of Y-shaped DNA–Ag nanoclusters (DNA-AgNCs), and also developed a hydrogel for wound healing [85]. The Y-shaped DNA-AgNCs (named Y-Ag) were synthesized, starting from three oligonucleotide strands (to form the Y structure) in the presence of NaBH_4_ and AgNO_3_. Hydrogel formation was then promoted by the addition of the same concentration of the other two oligonucleotide strands to introduce linkers. The Y-Ag composites’ formation was verified by electrophoresis and absorption spectroscopy. Bacteria inhibition against *E. coli* and *B. subtilis* was studied with colony forming units (CFUs), using PI and SYTO9 as the fluorescent labels for dead and live bacteria. Y-Ag had very good antibacterial activity, comparable to other AgNCs [86].

Y-Ag hydrogel, characterized by SEM, was tested with two methods: with bacteria grown on the surface of the gel and inside the gel. In both cases, the antibacterial properties were maintained and were improved when the Y-Ag concentration in the hydrogel was increased. Finally, with regard to an application in wound healing, good antibacterial activity was also reported for Y-Ag spray applied to a mouse skin wound model. DNA-synthesized AgNPs were also investigated in the work by Yang and collaborators [87]. These small NPs, also called silver nanoclusters, usually have, thanks to their very small sizes, luminescence properties [88], which are usually difficult to exploit because of their low stability. Their modification by DNA, other than making the AgNPs more stable, can tune their luminescence, such as in the case of the guanine-rich DNA strain, due to an electron transfer between guanine and the nanocluster [89,90]. In this regard, Yang et al. synthesized silver nanoclusters using a guanine-rich DNA strain linked to a bacterial (*S. aureus*) aptamer (AgNPs/Apt-G); a bacterial aptamer allows quenching the fluorescence. Once the aptamer entered into contact with the bacteria, disassembly was caused, which separated the guanine-rich sequence from the nanocluster and, thus, generated an on/off signal, making this system a sensor. Moreover, the presence of the aptamer promoted the interaction with the bacteria, enhancing the antibacterial activity. The synthesis of this system was performed in the presence of AgNO_3_, the DNA sequences and NaBH_4_ as the reducing agent. The AgNPs were characterized by TEM, and uniform nanoclusters of 1–3 nm were obtained. Fluorescence spectroscopy was used to choose the best number of complementary sequence bases, based on the highest fluorescence enhancement. The antibacterial activity was evaluated against Gram-negative *E. coli* and Gram-positive *S. aureus*, using MIC, and against *S. aureus*, using the fluorescence intensity of the AgNPs/Apt-G sensor. Good antibacterial activity was reported, which was better in the case of *E. coli* than in *S. aureus*, reflecting the trend already reported in the literature of the higher strength of Ag nanosystems towards Gram-negative, rather than Gram-positive, bacteria. Moreover, for *S. aureus*, the antibacterial effect was compared between AgNPs with and without the aptamer, showing that the presence of aptamers improves the performance, thanks to a more effective interaction between the nanoclusters and the bacteria. Then, the nanoclusters were electrospun with PLA to obtain a biodegradable fluorescent PLA-AgNPs nanofilm, whose antibacterial properties were confirmed to be suitable for future application in biomedical engineering and food packaging.

Beyond DNA, there are many other possibilities to functionalize silver nanoparticles with biomolecules, such as using antibiotics [91,92,93], amines [94,95], chitosan [96], polymeric materials [97,98], glycolipids [99], amino acids [100,101] and peptides [102,103]. For example, Aboelmaati and co-workers loaded green-synthesized AgNPs with two antibiotics, moxifloxacin and gatifloxacin, for antibacterial activity against bacterial biofilms [104]. The minimum biofilm inhibitory concentration against *Klebsiella pneumoniae*, *Pseudomonas aeruginosa* and *Acinetobacter baumannii* was evaluated. The synergy between the AgNPs and antibiotics resulted in an enhancement of the biofilms’ inhibition, compared to the poor antibiofilm activity of the separate AgNPs and antibiotics. Alternatively, Yang et al. used chitosan as the AgNPs functionality, obtaining a chitosan/carboxymethyl chitosan/silver nanoparticles (CTS/CMCTS/AgNPs) composite hydrogel [105]. The antibacterial activity, evaluated against *S. aureus* and *P. aeruginosa*, combined with the hydrogel biocompatibility, makes this material suitable for application in wound dressing.

In summary, among functionalization groups, recently there is a high focus on the use of biomolecules as reducing/capping agents. On one side, the use of plant extracts and bacteria cultures point to the synthesis of AgNPs utilizing green reagents as the source for the molecular components (polyphenols, flavonoids, etc.), which can reduce Ag ions and stabilize the nanoparticles. A drawback of this approach is surely the control of the size of the nanoparticles during the synthesis, which, in most cases, results in populations with a range of sizes. However, good antibacterial activity can be obtained. On the other side, DNA can be used for the functionalization of AgNPs, and in this case, even if the synthetic process is not completely green (NaBH_4_ is always used as the reducing agent), a higher control of the nanoparticles’ size is achieved. Indeed, under these conditions, nanoclusters are usually obtained, and, as described previously, additional properties can be included, such as fluorescence and target specificity. Additionally, many other biomolecules, such as antibiotics, amines, amino acids, peptides and polymers, can also be used as functionalized agents, leading to different beneficial effects. In conclusion, the production of AgNPs with plant/bacteria extracts, DNA or other biomolecules always enhances their biocompatibility, a property that is essential for biomedical engineering applications.

## 5. Antibacterial Silver Nanoparticles: Towards the Application

**Figure 6:**
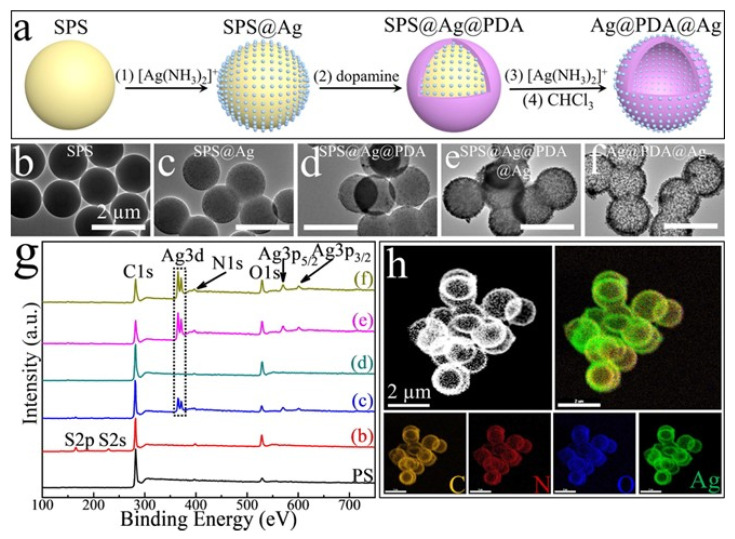
(**a**) Synthetic procedure of Ag@PDA@Ag NPs. (**b**–**f**) TEM images of the synthesis steps. (**g**) X-ray photoelectron spectroscopy of the synthetic steps. SPS (**b**); SPS@Ag (**c**); SPS@Ag@PDA (**d**); SPS@Ag@PDA@Ag (**e**); and Ag@PDA@Ag (**f**); (**h**) Dark-field TEM and energy-dispersive X-ray spectroscopy-based elemental mapping analysis of Ag@PDA@Ag NPs. All the scale bars are 2 μm. Reprinted with permission from ref. [106].

Among the requirements that antibacterial agents should possess, stability and controlled release of an active substance are very important, especially for applications in wound healing and in facing bacteria resistance. In view of this, a lot of silver-based composite materials have been developed where Ag nanoparticles are embedded in scaffolds, such as gels or films. An example of a composite system in which Ag nanoparticles can be embedded is given by Shang and collaborators [106]. In their work, they introduced AgNPs in a sandwich structure made of a polydopamine (PDA) shells, found both in the internal and in the external shells. In this way, it was possible to form a system that could quickly release AgNPs and, at the same time, thanks to the protection of the AgNPs in the internal shell, obtain durable antibacterial activity. In particular, they first synthesized sulphated polystyrene (SPS) colloidal particles that were then coated with Ag nanoparticles, obtained by Ag(NH_3_)_2_^+^ reduced and stabilized by PVP. Afterwards, by the addition of dopamine, a PDA coating was formed; the PDA coating was then decorated with AgNPs, obtained by the reduction of Ag(NH_3_)_2_^+^ performed by the PDA catechol and amino groups. Finally, the SPS was removed using trichloromethane so that a sandwich structure silver–polydopamine–silver (Ag@PDA@Ag) could be formed (Figure 6a). The nanoparticles were characterized by TEM, X-Ray spectroscopy and elemental mapping, which confirmed the presence of silver internally and externally (Figure 6b–h).

**Figure 7:**
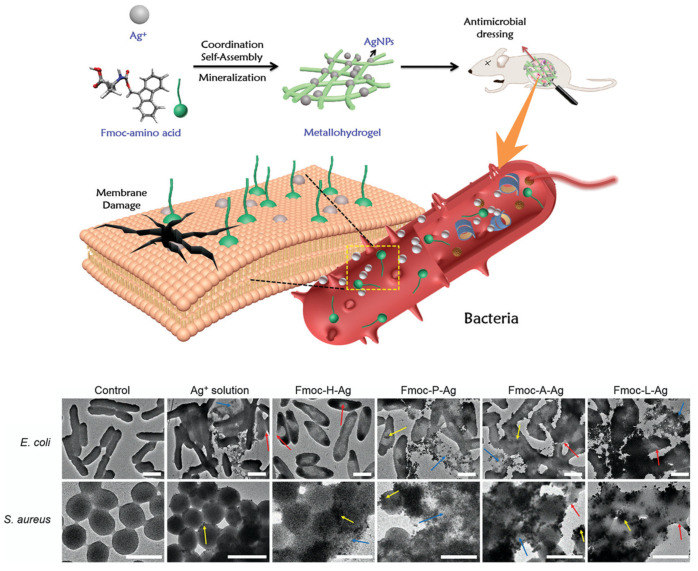
Formation of Ag^+^-coordinated Fmoc-amino acid metallohydrogels for application in antimicrobial dressing. Amphiphilic amino acids, AgNPs and Ag^+^ are able to interact and disrupt the bacteria membrane. Comparison between bacteria untreated and treated with different metallohydrogels and Ag^+^ solution. Red, yellow and blue arrows indicate membrane fusing, clumping and disintegration. All the scale bars are 1 μm. Adapted with permission from ref. [107].

The antibacterial activity of the system was evaluated against *E. coli* and *S. aureus* by means of Lysogeny broth and Mueller–Hinton broth agar plates, showing higher antibacterial performances for Ag@PDA@Ag rather than Ag@PDA and PDA@Ag in terms of activity and durability. In order to control the silver release in a biocompatible system, gels are usually exploited. For example, Song et al. reported the synthesis of biometallohydrogels based on the coordination and self-assembly of Ag^+^ and Fmoc-amino acids [107]: mixing Fmoc-amino acids and Ag^+^ solutions (in the presence of tris (hydroxymethyl) aminomethane-HNO_3_ as the reductant) leads to the formation of a gel, thanks to the coordination interaction between the silver ion and the amino acid. TEM and XPS results confirmed the presence of 6 nm AgNPs that were distributed along the hydrogel fibres, preventing their aggregation (Figure 7). The stability of the nanoparticles was also significantly improved by their binding with the C=O groups of the Fmoc-amino acids [108]. Moreover, their antibacterial activity was studied by means of disk diffusion methods, MIC and OD, comparing AgNPs gels prepared with different Fmoc-amino acids (Fmoc-His/Leu/Pro/Ala) and Ag^+^ solution as a reference. The biometallohydrogels presented higher antibacterial activity than Ag^+^ against both the Gram-negative *E. coli* and Gram-positive *S. aureus*, while changes in the bacterial morphology further demonstrated the ability of the biometallohydrogels to interact and disrupt the bacteria membranes (Figure 7). Furthermore, a high antibacterial activity was reported in Fmoc-Leu/Pro/Ala for Gram-positive bacteria, which are usually more resistant than Gram-negative ones. This could derive from the binding capability of some Fmoc-amino acids (such as Fmoc-Leu) to the *S. aureus* cell walls, aiding the interaction with the bacteria, despite the stiffness of their cell wall [109].

Finally, after assessing the biocompatibility of the biometallohydrogel, its wound healing performance was tested in wounds of mouse skin, further confirming the results obtained in vitro. In another case, Huang et al. developed an interesting system that was specific for the antibacterial treatment of burn wounds. In this case, a AgNPs- and gelatine-based cryogel (GT/Ag cryogel) was exploited. The idea consisted of the use of an antibacterial, biodegradable and porous GT/Ag cryogel that, thanks to its swelling ability, could also absorb wound exudate and, thus, permit gas exchange [110]. First, they prepared GT cryogel using EDC/NHS (1-(3-Dimethylaminopropyl)-3-ethylcarbodiimide hydrochloride/N-hydroxysuccinimide) as catalysers of the amidation reaction between the carboxylic and amino groups of GT. AgNPs were obtained with AgNO_3_ and sodium citrate, and then they were loaded into the cryogels by immersing them in a AgNPs solution.

Regarding the results, TEM indicated the presence of AgNPs of 10–20 nm, while XPS and SEM showed the porous structure of the cryogel, which also presented good mechanical properties (verified by the dynamic compression–strain test). An important aspect to evaluate was the shape-memory properties of the gel, which are an added value in the optics of wound healing. Shape-memory materials can be compressed in order to be injected in the wound, and then they expand, sealing the wound and preventing bleeding. GT/Ag cryogel resulted in being a good shape-memory material; the tested measuring swelling ratio, recovery time in water and volume expansion ratio, as well as the addition of AgNPs, did not affect its behaviour. Importantly the biocompatibility of GT/Ag was verified, and its antibacterial activity was tested, by means of OD measurements, against *E. coli*, *P. aeruginosa* and methicillin-resistant *S. aureus*, the latter two of which are particularly favoured in burn wounds.

Good antibacterial activity was reported for GT/Ag cryogel, even against bacterial biofilm. In vivo tests were also performed, showing that GT/Ag cryogel had a higher capability to absorb burn wound exudates than commercial wound dressings. Another proper material for application in wound dressing is electrospun nanofibers, thanks to their surface and porosity. Yang et al. proposed electrospun Janus fibres with loadings of an antibiotic (ciprofloxacin—CIP) and AgNPs [111]. Janus fibres are made with independent and parallel parts in the micro–nano scale and are composed of a side-by-side membrane with electrospun layers [112]. In this work, PVP and ethyl cellulose (EC) polymers, representing the two layers, were electrospun with CIP and AgNPs in the two sides, respectively. The fibres’ morphology was characterized by SEM and TEM, which showed the presence of AgNPs. The antibacterial activity, tested with the disk diffusion method, displayed good results for both *E. coli* and *S. aureus*. In particular, CIP/AgNPs fibres showed better performances than CIP fibres or AgNPs fibres, synthesized for comparison, indicating the synergistic action of the antibiotic and the silver. Other scaffolds that have biomedical applications are cubosomes, self-assembled lipidic colloidal particles of lyotropic liquid crystalline phases [113], with a cubic morphology. Meikle and co-workers functionalized cubosomes with metal nanocrystals, such as AgNPs, in order to obtain a delivery system for antimicrobial activity [114]. The system was obtained by dissolving Ag nanocrystals, previously prepared using oleic acid as a capping agent, and lipids (monoolein or phytantiol) in heptane; the solvent was then removed in vacuo, and Pluronic F-127 was added to the mixture as a stabilizing agent. The Ag nanoparticles had a size of almost 3.5 nm (observed by TEM), and after being embedded in cubosomes, were characterized by cryo-TEM, which showed the presence of associated nanocrystals in the cubosomes. The cubosomes’ antimicrobial behaviour was assessed against *S. aureus*, *P. aeruginosa*, *B. cereus* and *E. coli* by MIC, resulting in generally good antibacterial activity that was higher in the Gram-negative than in the Gram-positive species. This is expected due to the higher resistance of Gram-positive bacteria, and it is also correlated with the Gram-negative external lipid membrane that was able to favour the cubosomes’ adsorption and subsequent release of AgNPs. The mechanism of AgNPs’ delivery was also investigated by total internal reflection fluorescence microscopy (staining cubosomes with a cyanine dye) on *S. aureus* and *B. cereus*, which confirmed the interaction between the bacteria cells and cubosomes.

Recently, there have been more and more attempts to embed AgNPs in matrixes that are biocompatible, biodegradable, easy to apply and have to provide a controlled release of AgNPs. In particular, it is important to have a local and controlled, in terms of time, release. This kind of delivery allows minimizing toxicity and amplifying the AgNPs’ effects, even in drug-resistant bacteria.

## 6. Size, Shape and Functionalization Effects on Cytotoxicity

The antibacterial properties of AgNPs make them suitable for many applications in the biomedical field, food packaging and personal care [115]. Considering their widespread use, it is important to understand which is the toxicity of silver nanomaterials. Even if the mechanisms involved in the AgNPs’ interaction with cells is not completely elucidated, their physical and chemical properties surely have effects on their toxicity [116,117,118,119], and these effects need to be investigated. An example of size-dependent AgNPs’ toxicity is clearly observed in the study of Gliga et al., which evaluated the effect of AgNPs on the immune response and distinguished the impact of two populations: 10 nm and 75 nm nanoparticles [120]. Their tests, performed on monocyte, macrophage and lung epithelial cells, highlighted that 10 nm nanoparticles were more cytotoxic than 75 nm nanoparticles and that 10 nm nanoparticles were also immunosuppressive. Moreover, they observed, by means of inductively coupled plasma mass spectrometry (ICP-MS), a higher silver release in the cellular medium in the case of 10 nm nanoparticles rather than in the 75 nm ones. Auclair and co-workers investigated the toxicity of AgNPs based on their shape [121]. They investigated the behaviour of spherical, cubic and prismatic nanoparticles in the presence of the invertebrate *Hydra vulgaris*. In particular, they found the spherical nanoparticles to be more toxic than the cubic and the prismatic ones. This result is in contrast with some studies on this topic [122,123,124] and in line with others [125], showing how the shape effect is still not clear, because in many cases, it is coupled with the effects of size and surface functionalities. Indeed, surface functionalization also has an impact on the toxicity of AgNPs. For example, O’cwieja et al. investigated the cytotoxicity of AgNPs stabilized by cysteine moieties in HL-60 and U-937 cell lines [126]. In particular, they found that the toxicity of cysteine functionalized AgNPs was mainly given by mitochondrial damage, which they attributed to the positively charged cysteine functionalities. A higher toxicity correlated with positively charged surface groups of AgNPs was also found in other studies, such as the one by Vukovic et al., which compared the toxicity effect of neutral polymer poly(vinylpyrrolidone), positively charged poly-L-lysine and negatively charged bis(2-ethylhexyl) sulfosuccinate sodium as AgNPs’ stabilizers [127]. The physical and chemical properties of AgNPs have been shown to strongly influence their cytotoxicity. As a consequence, the design of antibacterial AgNPs needs to consider in parallel the possible toxicity effects to avoid risks for human health.

## 7. Conclusions

In this review, AgNPs’ properties and modifications were correlated with the effectiveness of antibacterial activity. The main property of an antibacterial agent is its capability to interact with and modify the bacterial external membrane, resulting in an easier internalization. For AgNPs, the size effect showed the importance of having sufficiently small nanoparticles to allow an efficient cellular uptake. The shape’s role in antibacterial activity is more controversial because it is usually coupled with the size effect. Generally, the antimicrobial performance is improved in the presence of sharp edges, corresponding to more reactive parts, and depending on the exposed crystal facets.

Apart from size and shape, another attempt to optimize the penetration in bacteria, with subsequent damage, is the surface functionalization. This is usually performed with moieties that facilitate interaction with bacteria, such as the specific binding with recognition sites in the bacteria surface, as in the case of DNA/aptamer functionalities. Other important features to bear in mind in the design of antibacterial AgNPs are the biocompatibility of the nanosystem and the capability to have an attack amplification against the cell by the simultaneous release of reactive species or the synergistic contribution of different antibacterial agents. Embedding AgNPs in other materials, such as gels and polymers, can help to control/amplify nanoparticles’ release. In summary, in order to best optimize AgNPs’ efficacy for future application, it is important to not only design the systems in all their properties based on the specific targets, but also to obtain a biocompatible system, minimizing the toxicity of AgNPs.

## 8. Future Perspectives

Even if there are many strategies for the modification and adaptation of AgNPs against bacterial infections, this work is focused on specific characteristics, which are their size, shape, functionalization and modification by other materials. Considering these parameters, we found that there are still some issues to solve for the design of AgNPs with the right characteristics against MDR.

### 8.1. Size and Shape Comparison

Size and shape are very important parameters because they represent intrinsic properties of AgNPs, without the influence of any functionalization or modification. While the correlation between size and activity is quite clear, there are still no certainties regarding the shape influence; looking through the literature, the trend of activity and morphology is not always correlated in the same way. Indeed, often a control on the nanoparticles’ shape is not accompanied by a size control, and therefore, a comparison is made between nanoparticles not only with different shapes, but also with different sizes. On the contrary, in order to have a precise association between morphology and activity, it would be necessary to always synthesize different-in-shape nanoparticles with the same size. In this way, it would be possible to exclude size effects that are probably predominant with respect to the shape effects.

### 8.2. Role of Functionalities in the Antibacterial Mechanism

Concerning AgNPs’ functionalization, there are now many synthetic opportunities, and research is particularly focused on functionalization by biomolecules obtained from bacteria or plant extracts. Every time a natural, complex chemical mixture is used, many biomolecules can be involved, and often, their precise function is not clear in the antibacterial activity. Individuating the exact role of each functional group, which can be obtained by bacteria or plant extracts, could be a good strategy to rationalize AgNPs’ functionalization strategy and optimize their behaviour.

### 8.3. Standard Methods to Evaluate Antibacterial Activity

In the literature, antibacterial activity is usually tested against a group of pathogens by means of some tests chosen by the authors. Nevertheless, in this way, it is difficult to really correlate the AgNPs’ efficiency between the different methods described in various works. Having a standardized technique based on a defined group of bacteria, Gram-negative and Gram-positive, and on the same testing protocols, in terms of methods, concentrations, etc., would be an upgrade as a comparison tool. Moreover, a standard protocol would also be useful to understand which one of the AgNPs’ properties, i.e., size, shape or functionalization, is prevalent in the antibacterial behaviour.

Considering all these aspects and improving them could allow obtaining more controlled AgNPs that can be designed based on their functions in specific applications.

## Data Availability

Data sharing not applicable.
